# Evaluation and optimization of a conventional SPCE for FMD post-vaccination monitoring

**DOI:** 10.1186/s12917-018-1686-7

**Published:** 2018-11-28

**Authors:** Yeong-Lim Kang, Ji-Yun Jeong, Hwi-Yeon Choi, Yanhong Zhang, Yumei Liu, Ho-Jong Lee, Jong-Chul Choi, So-Hyun Lee, Beom-Joo Lee, Sang-Won Lee, Joong-Bok Lee, Ki-hyun Cho, Seung-Yong Park

**Affiliations:** 1 Laboratory of Veterinary Immunology, College of Veterinary Medicine, Konkuk University, 120 Neungdong-ro, Gwangjin-gu, Seoul, 05029 Republic of Korea; 2 Jinyu Baoling Bio-pharmaceutical Co., Ltd., Hohhot, 010030 Inner Mongolia China; 3 Veterinary Epidemiology Division, Animal and Plant Quarantine Agency, 177, Hyeoksin 8-ro, Gimcheon, Gyeongsangbuk-do 39660 Republic of Korea

**Keywords:** Foot-and-mouth disease, Serological monitoring, Vaccination coverage, Solid-phase competitive ELISA, Liquid-phase blocking ELISA, Virus neutralization test

## Abstract

**Background:**

Foot-and-mouth disease (FMD) can be controlled by either stamping out or vaccination, a choice which depends on both the economic importance of the livestock sector as well as the disease status. In FMD-free countries with vaccination, such as Korea, vaccination programs should guarantee prevention against transmission of FMD. Monitoring of vaccination programs is also essential for ensuring sufficient coverage that will limit the transmission of FMDV. There are several methods to screen FMD virus (FMDV) structural protein (SP) antibodies including SPCE (Solid-phase competitive ELISA), LPBE (Liquid-phase blocking ELISA), and VNT (Virus neutralization test). Among these, SPCE is widely used for serological monitoring since VNT—the gold standard method—has certain practical limitations, such as high costs in terms of time and labor. However, whether SPCE can ensure the vaccination status of individual animals and whole farms is unclear. In this study, SPCE, LPBE and VNT were compared with respect to correlation with each other and sensitivity at commercial pig farms.

**Results:**

The positive results obtained by PrioCHECK SPCE differed from those obtained by LPBE and VNT. The sensitivity of SPCE relative to those of the other tests was fairly low. The raw data of SPCE were most highly correlated with those of VNT with XJ strain, while their positivity and negativity were most highly correlated with LPBE. The results of ROC analysis proposed new cut-off for PrioCHECK SPCE higher than the previous 50% inhibition.

**Conclusions:**

The high false positive rate of PrioCHECK SPCE suggested that high seropositivity by SPCE may not guarantee a true vaccination coverage. Adjusting the cut-off percentage (%) inhibition value for SPCE is needed to address this problem, and it is highly recommended that routine FMDV serological monitoring programs using PrioCHECK SPCE should be combined with alternative methods such as LPBE or VNT.

## Background

Foot-and-mouth disease (FMD) is a viral disease caused by the FMD virus (FMDV), which belongs to the genus *Aphthovirus* in the Picornaviridae family [1]. The contagious nature of the virus and its various serotypes make it a major threat to animal husbandry worldwide [2]. Rapid and precise diagnosis is a prerequisite for preventing the spread of this disease. One diagnostic approach is to detect FMDV-specific antibodies; serological monitoring tests usually detect antibodies against either non-structural proteins (NSPs) [3–7] or structural protein (SPs). Solid-phase competition enzyme-linked immunosorbent assay (ELISA) (SPCE) [8–10], liquid-phase blocking ELISA (LPBE) [11, 12], and the virus neutralization test (VNT) [13] are typically used for SP antibody screening. They are performed in support of four main purposes: 1) to certify individual animals prior to import or export; 2) to confirm suspected cases of FMD; 3) to substantiate absence of infection; and 4) to demonstrate the efficacy of vaccination [14].

In FMD-free countries with vaccination, such as Korea, SPCE is adopted as a screening method for evaluating herd immune status after FMD vaccination because the gold standard method, VNT, is time and labor consuming. In Korea, there is a cut-off value of vaccination coverage by SPCE according to the relevant regulations, and farms with less than this value are subject to a fine. Despite these efforts, FMD has still been detected in premises with sufficient vaccination coverage above the cut-off value. Moreover, it is unclear whether SPCE is an appropriate method to certify individual animals and whether it demonstrates efficacy for the evaluation of vaccination status.

In the present study, we investigated whether PrioCHECK SPCE is appropriate for determining the FMD vaccination status of farms by comparing the vaccination coverage and correlation to those obtained by other SP antibody test methods and by assessing the relative sensitivity and specificity of the assay.

## Results

### SP antibody response after vaccination

To compare the performance of the three SP antibody tests, we evaluated vaccination coverage and antibody response based on logarithmic titers determined by each assay. Results of LPBE were confirmed by performing the assay using reagents from the World Reference Laboratory for Foot-and-Mouth Disease (data not shown).

**Fig. 1:**
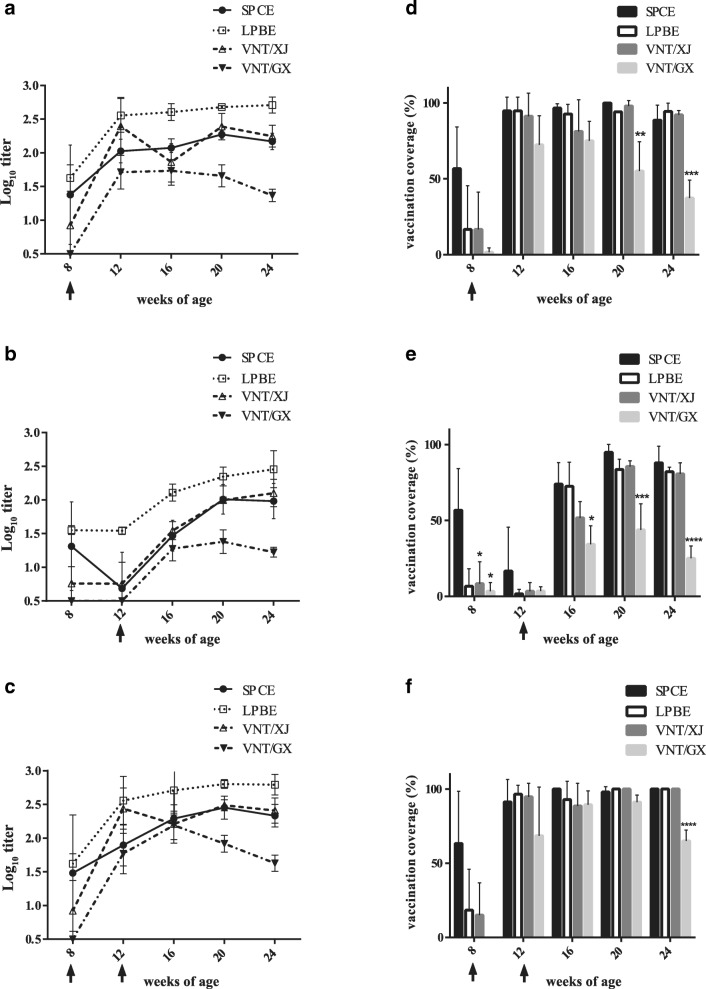
**a**–**f** Progression of antibody response (**a**–**c**) and vaccination coverage (**d**–**f**) determined by SPCE, LPBE, and VNT. Data for group I (**a**, **d**), group II (**b**, **e**), and group III (**c**, **f**) are shown. The arrow indicates the timing of vaccination. Results of SPCE are expressed as PI, while those of LPBE and VNT are expressed as a logarithmic titer. Criteria for positivity by SPCE, LPBE, and VNT are 50%, 1/64 (1.81 log_10_), and 1/45 (1.65 log_10_), respectively. The results of average titer are presented as mean ± SD (*n* = 60). *P < 0.05, **P < 0.01, ***P < 0.001

The development of SP antibody was compared among the three methods used in the study (Fig. 1a–c). In the case of SPCE, the antibody level was highest 12 weeks after the vaccination in group I (Fig. 1a), whereas the level peaked after 8 weeks in group II (Fig. 1b). In group III, the antibody response was highest 8 weeks after the second vaccination (Fig. 1c).

Interestingly, the percentage of positive reactions before vaccination was above 50% when tested by SPCE, but it was below 20% when tested by LPBE and VNT in all groups (Fig. 1d–f). No NSP antibody was detected in any of the samples (data not shown). However, there were no significant differences between LPBE or VNT with XJ strain (VNT/XJ) and SPCE titers except for group II at 8 weeks of age. SPCE differed significantly from VNT/GX at 20 and 24 weeks of age in group I, at 8, 16, 20 and 24 weeks of age in group II, and at 24 weeks of age in group III. At market age, the mean vaccination coverage of SPCE were 88.56, 87.98 and 100% for each group.

### Scatter plots and calculated regression lines for each assay

**Fig. 2:**
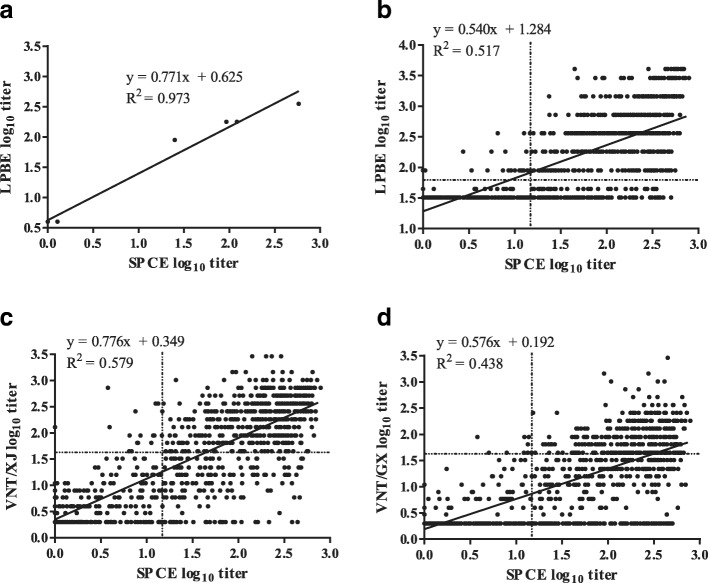
**a**–**d** Scatter plots and calculated regression lines between SPCE and LPBE (**a**, **b**) and between SPCE and VNT (**c**, **d**). Scatter plots show PI ratio of SPCE on the x-axis and logarithmic titer of LPBE or VNT on the y-axis. Dotted line represents the cut-off value for each test (1.17, SPCE; 1.81, LPBE; and 1.65, VNT)

To further investigate the correlation between PrioCHECK SPCE and other assays and obtain regression lines, scatter plots were generated that showed logarithmic titers of SPCE on the x-axis and those of LPBE or VNT on the y-axis. The results for four positive and three negative reference serum samples obtained by SPCE were highly correlated with those obtained by LPBE (R^2^ = 0.973, *P* < 0.0001) (Fig. 2a). However, not all the field samples that tested positive by SPCE were positive by LPBE (R^2^ = 0.517) (Fig. 2b). Nevertheless, we observed a trend that all but seven samples, positive by LPBE, had a log_10_ titer > 0.85 by SPCE. On the contrary, LPBE titers of samples positive by SPCE ranged from 1.51 to 3.61.

In the scatter plots for SPCE and VNT, R^2^ was higher when SPCE was matched with VNT/XJ (R^2^ = 0.579) than when it was matched with VNT/GX (R^2^ = 0.438) (Fig. 2c, d). Most serum samples positive by VNT had a log_10_ titer > 0.85 by SPCE, except six and two samples of the XJ and GX strain, respectively. On the other hand, VNT titers of sera positive by SPCE ranged from 0.3 to 3.46.

### Correlation and relative sensitivity of SPCE compared to other tests

We calculated the Pearson correlation coefficient between PrioCHECK SPCE and other SP tests by comparing the data at the individual level. Overall, the highest correlation coefficient was between VNT/XJ and VNT/GX (*r* = 0.803, *P* < 0.001) (Table 1). SPCE showed the highest correlation with VNT/XJ (*r* = 0.761), followed by LPBE (*r* = 0.719) and VNT/GX (*r* = 0.662).

**Table 1:** The Pearson correlation among SP antibody test methods

	SPCE	LPBE	VNT/XJ	VNT/GX
SPCE	1	0.719	0.761	0.662
LPBE		1	0.783	0.777
VNT/XJ			1	0.803
VNT/GX				1

*All correlation values are significant at a confidence level of 0.001 (2-tailed)

The relative sensitivity and specificity of SPCE were compared with LPBE, VNT/XJ and VNT/GX (Table 2). A total of 25, 19, and 9 samples confirmed positive by LPBE, VNT/XJ, and VNT/GX, respectively, were negative by SPCE. Conversely, 147, 164, and 342 samples positive by SPCE were negative by LPBE, VNT/XJ, and VNT/GX, respectively.

**Table 2:** Comparison of sensitivity and specificity of SPCE with LPBE, VNT/XJ and VNT/GX

Method		LPBE		VNT/XJ		VNT/GX	
	Result	Positive	Negative	Positive	Negative	Positive	Negative
SPCE	Positive	531	147	514	164	336	342
	Negative	25	251	19	257	9	267
Cohen’s kappa		0.612		0.596		0.340	
Overall concordance (%)		82.0		80.8		63.2	
False positive (%)		21.7		24.2		50.4	
False negative (%)		9.1		6.9		3.3	

SPCE showed the highest concordance with LPBE, followed by VNT/XJ and VNT/GX (Table 2). Cohen’s kappa of SPCE was highest with LPBE. False negative rates were less than 10%. However, the false positive rate was more than 20%, and in case of SPCE with VNT/GX, it was 50.4%. It means that more than half of pigs identified as positive by SPCE screening did not have sufficient antibodies for protection.

### Standardization of cut-off value for SPCE

**Fig. 3:**
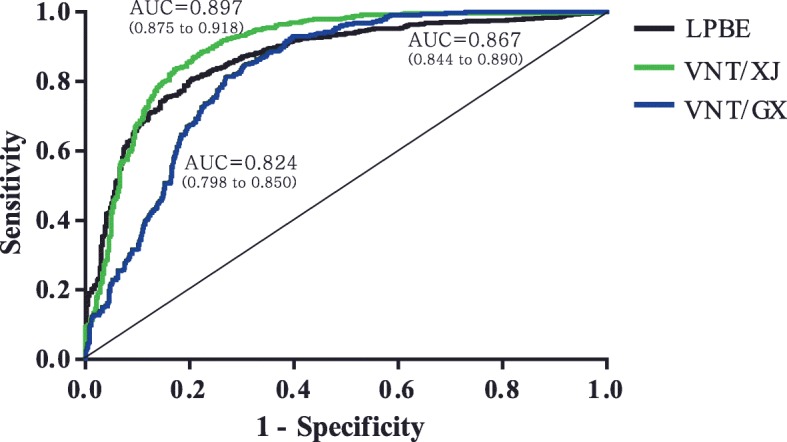
ROC curve analysis of results of individual SP antibody tests. The AUC is shown for each graph, with the 95% confidence interval in parentheses

To decrease false positive rate of PrioCHECK SPCE, we determined the optimal cut-off for PI of SPCE in 1/10 dilution and generated receiver operated characteristic (ROC) curves using the results obtained by other SP tests as standards (Fig. 3). Because the results of PrioCHECK SPCE were expressed as PI in general, PI results, not log_10_ titer, were used in this time. The area under the curve (AUC), sensitivity, and specificity were also calculated, and the optimal cut-off value was determined to obtain the maximum sensitivity and specificity.

The AUC was largest when the standard was VNT/XJ, followed by LPBE and VNT/GX (*P* < 0.0001) (Fig. 3). The optimal cut-off for SPCE was 63.55% when compared with LPBE, with a sensitivity of 0.801 and specificity of 0.800. For VNT/XJ, the cut-off value was 67.23% and the sensitivity and specificity were 0.834 and 0.829, respectively. For VNT/GX, the cut-off value was 76.83% and the sensitivity and specificity were 0.753 and 0.753, respectively.

## Discussion

In this study, we compared PrioCHECK SPCE with LPBE and VNT in terms of their potential to demonstrate efficacy for the evaluation of vaccination status at the herd level and certification of animals at the individual level. All these methods detected SP antibodies generated 5–8 days after FMDV infection or vaccination in pigs [15]. However, there was disparity among the results, possibly due to the antigen used in each assay, that is, the vaccine strains O/Mya-98/XJ/2010 and O/Cathay/GX/09–7 for VNT and O/SEA/Mya-98 for LPBE.

We expected a high correlation between LPBE and VNT since the former was considered a replacement assay for the latter [16]. A high correlation coefficient proved this to some degree.

The antibody responses measured by SPCE are consistent with those reported in previous studies [17, 18]. More specifically, the vaccination coverage of group I was higher than that of group II at market age. Additionally, both groups had the highest vaccination coverage at 20 weeks of age. These results, along with previous findings by our group and others [17, 19], suggest that vaccination at 8 weeks of age achieves the highest vaccination coverage as detected by SPCE.

The antibody titer and vaccination coverage determined by SPCE were relatively similar to those determined by LPBE and VNT at 4–12 weeks after vaccination and differed from the values measured before and 16 weeks after vaccination. The significant difference in the results obtained during the late period of vaccination poses a potential threat since SPCE was performed immediately before the animals were taken to the market and vaccination is usually performed only once from 8 to 12 weeks of age in many countries. Moreover, vaccination coverage by SPCE was above 50% even before the vaccination. It suggests that SPCE is less specific than other SP test methods since there was no infection during the study, supported by the absence of NSP antibody.

One of the characteristics of PrioCHECK SPCE was the high false positive rate and relatively low correlation coefficient compared to the other test methods. As a general rule of thumb, specific tests are needed to rule in diagnoses, and highly sensitive tests are needed to rule them out. [20] Thus, the results of SPCE do not fully reflect the protective capacity of sera, unlike LPBE and VNT, which measure the neutralizing capacity. In addition, a high false positive rate can be a serious problem in FMD-endemic countries, given the highly contagious nature of FMDV.

Our study showed that almost 60% of the serum samples were positive for PrioCHECK SPCE before vaccination. Therefore, 50% inhibition is inadequate as a cut-off value; the results of the ROC analysis suggested 63.55% inhibition as a minimum value. We also found that 1:10 is the optimal dilution for PrioCHECK SPCE because there was no overlap between positive and negative sera (data not included). It was demonstrated previously that 30% inhibition at 1:5 dilution was the optimal cut-off for differentiating positive from negative serum [9], and that 60% inhibition was a proper cut-off for SPCE [10]. Further studies are needed to establish a definitive standard.

## Conclusions

This study evaluated and optimized PrioCHECK type O SPCE for post-vaccination monitoring. The positive results obtained by SPCE were higher than those obtained by LPBE and VNT, especially at 8 weeks of age. SPCE showed a relatively high false positive rate compared with LPBE and VNT, suggesting that positivity by PrioCHECK SPCE is not sufficient to certify animals and to confirm the implementation of vaccination. Adjusting the cut-off PI value for SPCE may be a possible solution to this problem. The raw data of PrioCHECK SPCE had the highest correlation with VNT/XJ, while the positivity and negativity were highly correlated with LPBE. Nevertheless, correlations between SPCE and other tests were lower than those among other tests. It is highly recommended that routine FMDV post-vaccination monitoring and corresponding regulatory programs using PrioCHECK SPCE should be combined with alternative methods such as LPBE or VNT.

## Methods

### Animals

The pigs used in this study were kept on three farrow-to-finish commercial farms in Pocheon, Gyeonggi province. Routine vaccination programs for porcine circovirus type 2 virus, classical swine fever virus, FMDV, and *Mycoplasma hyopneumoniae* were carried out. Infectious diseases such as porcine epidemic diarrhea and FMD were not detected. On each farm, 60 piglets were raised and selected at the age of 7 weeks; these pigs were divided into three groups based on the results of PrioCHECK SPCE antibody level against FMD type O. Each group consisted of 20 pigs for each farm and the average antibody levels were similar among the groups. After this study, all pigs of market age were sent to the abattoir and slaughtered humanely. They were stunned with electricity and bled without consciousness according to the Article 10 of Animal Protection Law of Republic of Korea.

### Vaccination and sample collection

Animals were vaccinated with inactivated FMD type O bivalent vaccine consisting of O/Mya-98/XJ/2010 and O/Cathay/GX/09–7 strains in oil adjuvant (Jinyu Baoling Bio-pharmaceutical Co., Hohhot, China). These strains were selected based on their genetic homology to field strains isolated in Korea and neighboring countries. The vaccine was formulated to contain at least six times the 50% protective dose.

A single dose (2 ml) of the vaccine was injected intramuscularly into group I (8 weeks old) and group II (12 weeks old). Group III was vaccinated twice at 8 and 12 weeks old. These schedules were designed to analyze antibody response after vaccination in the presence of maternally derived antibodies. Blood samples were collected at 8, 12, 16, 20, and 24 weeks of age in all three groups to evaluate SP antibody titers. The sample size fluctuated during the experimental period because some of the pigs died. Serum samples were stored at − 20 °C or − 70 °C until the analysis.

### SPCE

FMDV type O SP antibody levels were determined using the PrioCHECK FMDV Type O Antibody ELISA kit (Prionics AG, Schlieren-Zurich, Switzerland) according to the manufacturer’s instructions. PrioCHECK SPCE was chosen because it is most frequently adopted as a primary screening method in Korea. Briefly, a microtiter plate coated with FMD type O antigen was incubated with a 1/10 dilution of serum for 1 h at room temperature. After washing, a predetermined dilution of conjugate was added, and the plate was incubated for 1 h at room temperature. The plate was washed and the chromogenic substrate 3,3′,5,5′-tetramethylbenzidine (TMB) was added. The results were expressed as percentage inhibition (PI) relative to the maximum optical density at 450 nm. FMDV type O SP antibodies were considered to be absent and present in the serum if the PI was < 50 and ≥ 50%, respectively. Additionally, serum samples were 1:2 serially diluted and the 50% endpoint titers were calculated.

### LPBE

LPBE was performed using a commercial kit (Lanzhou Veterinary Research Institute, Lanzhou, China) according to the manufacturer’s manual. The assay is based on specific blocking of a defined amount of FMDV antigen by antibodies in the test serum in liquid phase. Briefly, a mixture of viral antigen (O/SEA/Mya-98) and diluted test serum was incubated overnight at 4 °C in liquid phase. The mixture was transferred to a trapping antibody-coated immune plate and incubated for 1 h at 37 °C. After washing, the plate was incubated with FMDV type O guinea pig antibody for 30 min at 37 °C. The plate was washed, rabbit anti-guinea pig IgG conjugated with horseradish peroxidase was added, and incubated for 30 min at 37 °C. After washing, the chromogenic substrate, TMB, was added. The results were expressed as the 50% endpoint titer, that is, the dilution at which the reaction of the test serum yields an optical density equivalent to half of the reaction (antigen) control wells. If the titer was equal to or above 1/64 (1.81 log_10_), samples were considered to be positive.

### VNT

Serum samples were tested for virus-neutralizing antibodies against FMDV vaccine strains (O/Mya-98/XJ/2010 and O/GX/09–7) according to the standard protocol of the Animal and Plant Quarantine Agency (Gimcheon, Korea). Two-fold dilutions of the sample were distributed across the plate, starting from a 1/2 dilution. Previously titrated virus was added within log_10_ 1.5–2.5 TCID_50_ per well. After 1 h incubation at 37 °C, LFBK cell suspension was added at 2.5 × 10^4^ cells per well. Plates were incubated at 37 °C for 3 days. Titers were expressed as the final dilution that inhibited virus growth in 100% of the wells. A titer of ≥1/45 (1.65 log_10_) and < 1/16 of the final serum dilution in the serum/virus mixture was considered positive and negative, respectively.

### Statistical analysis

Data are presented as mean ± standard deviation. The Pearson correlation coefficient was calculated using Prism v.6.0 software (Graph Pad Inc., La Jolla, CA, USA). Scatter plots were generated using the same program. Group means were compared by the one-way analysis of variance with multiple comparisons. Cohen’s kappa of the assay was calculated by comparing the number of true positives and true negatives. Receiver Operating Characteristic (ROC) curves that illustrate the diagnostic ability of assay were drawn. The optimal cut-off value and the sensitivity and specificity were determined using the ROC curves.

## Abbreviations

AUC: Area under curve
ELISA: Enzyme-linked immunosorbent assay
FMD: Foot-and-mouth disease
FMDV: FMD virus
LPBE: Liquid-phase blocking ELISA
NSP: Non-structural protein
PI: Percentage inhibition
ROC: Receiver operated characteristic
SP: Structural protein
SPCE: Solid phase competition ELISA
TCID: Tissue culture infective dose
VNT: Virus neutralization test
